# Engineering Synthetic Lipopeptide Antigen for Specific Detection of *Mycobacterium avium* subsp. *paratuberculosis* Infection

**DOI:** 10.3389/fvets.2021.637841

**Published:** 2021-04-23

**Authors:** Sylvie Bay, Douglas Begg, Christelle Ganneau, Maxime Branger, Thierry Cochard, John P. Bannantine, Heike Köhler, Jean-Louis Moyen, Richard J. Whittington, Franck Biet

**Affiliations:** ^1^Institut Pasteur, Unité de Chimie des Biomolécules, Département de Biologie Structurale et Chimie, Paris, France; ^2^CNRS UMR 3523, Paris, France; ^3^School of Veterinary Science, University of Sydney, Camden, NSW, Australia; ^4^INRAE, Université de Tours, ISP, Nouzilly, France; ^5^USDA-Agricultural Research Service (USDA-ARS), National Animal Disease Center, Ames, IA, United States; ^6^Friedrich-Loeffler-Institut, Federal Research Institute for Animal Health, Jena, Germany; ^7^Laboratoire Départemental d'Analyse et de Recherche de Dordogne, Coulounieix Chamiers, France

**Keywords:** *Mycobacterium avium* subsp. *paratuberculosis*, Johne's disease, lipopeptide, diagnosis, antibody response, chemical synthesis

## Abstract

Unlike other MAC members, *Mycobacterium avium* subsp. *paratuberculosis* (MAP) does not produce glycopeptidolipids (GPL) on the surface of the cell wall but a lipopentapeptide called L5P (also termed Lipopeptide-I or Para-LP-01) characterized in C-type (bovine) strains. This lipopeptide antigen contains a pentapeptide core, D-Phenylalanine-N-methyl-L-Valine-L-Isoleucine-L-Phenylalanine-L-Alanine, in which the N-terminal D-Phenylalanine is amido-linked with a fatty acid (C18–C20). The molecular and genetic characterization of this antigen demonstrated that L5P is unique to MAP. Knowledge of the structure of L5P enabled synthetic production of this lipopeptide in large quantities for immunological evaluation. Various studies described the immune response directed against L5P and confirmed its capability for detection of MAP infection. However, the hydrophobic nature of lipopeptide antigens make their handling and use in organic solvents unsuitable for industrial processes. The objectives of this study were to produce, by chemical synthesis, a water-soluble variant of L5P and to evaluate these compounds for the serological diagnosis of MAP using well-defined serum banks. The native L5P antigen and its hydrosoluble analog were synthesized on solid phase. The pure compounds were evaluated on collections of extensively characterized sera from infected and non-infected cattle. ROC analysis showed that L5P and also its water-soluble derivative are suitable for the development of a serological test for Johne's disease at a population level. However, these compounds used alone in ELISA have lower sensitivity (Se 82% for L5P and Se 62% for the water-soluble variant of L5P) compared to the Se 98% of a commercial test. Advantageously, these pure synthetic MAP specific antigens can be easily produced in non-limiting quantities at low cost and in standardized batches for robust studies. The fact that L5P has not been validated in the context of ovine paratuberculosis highlights the need to better characterize the antigens expressed from the different genetic lineages of MAP to discover new diagnostic antigens. In the context of infections due to other mycobacteria such as *M. bovis* or the more closely related species *M. avium* subsp. *hominissuis*, the L5P did not cross react and therefore may be a valuable antigen to solve ambiguous results in other tests.

## Introduction

**Figure 1:**
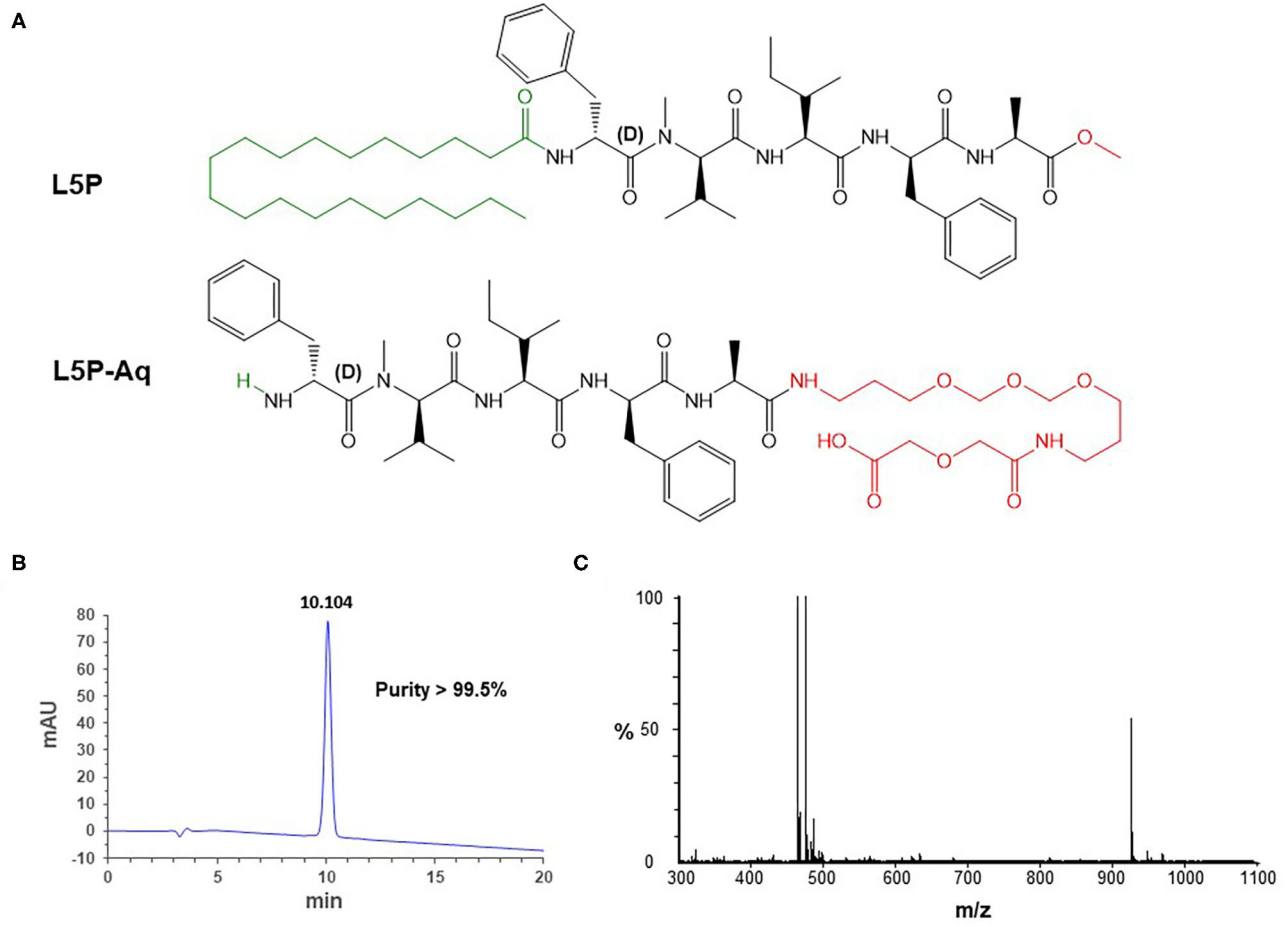
Structural formulas of L5P and L5P-Aq **(A)**. The amino-acid residues belong to the L-series, unless otherwise specified. The N-ter and the C-ter moieties are indicated in green and red, respectively. Physico-chemical characterization of the purified synthetic L5P-Aq by RP-HPLC **(B)** and MS **(C)**. **(B)** Detection is performed by UV at 230 nm (milli-absorption units). Purity is indicated in % area-under-the-curve. Retention time is indicated above the peak in min. **(C)** Mass/charge values in daltons: *m/z* 464.773 [M+2H]^2+^ (calcd 464.774), *m/z* 475.754 [M+H+Na]^2+^ (calcd 475.765), *m/z* 928.555 [M+H]^+^ (calcd 928.540), *m/z* 950.514 [M+Na]^+^ (calcd 950.522). See Supplementary Figure 1 for NMR spectra.

Mycobacteria are well-known for their cell envelope containing abundant mycolic acids and specific lipid components (1, 2). These molecules play a crucial role to maintain the integrity of the cell wall and are considered to be involved in bacterial virulence through influence on the host immune response (3). The outer membrane contains diverse surface lipids that are species-dependent. This likely reflects differences in the cell biochemical organization and probably impacts significantly the way *Mycobacterium* species interact with the host. *Mycobacterium avium* complex (MAC) is a group of non-tuberculous mycobacteria that cause tuberculosis-like diseases in humans and in animals and that produces glycopeptidolipids (GPLs) (4, 5). GPLs form the basis for a commercial test that can be useful for rapid diagnosis of MAC pulmonary disease (MAC-PD) and for differentiating MAC-PD from pulmonary tuberculosis (PTB) in humans (6). Although belonging to the MAC, the subspecies *paratuberculosis* (MAP), causing paratuberculosis or Johne's Disease in ruminants (7), is distinguished by the absence of production of GPL (8). Subspecies *paratuberculosis* produces lipopeptides (LPs) whose biosynthetic pathway is likely to be homologous to that of GPL, based on the structural relatedness between GPL and LP. The main difference between GPL and LP is the absence of sugars, explained by absence of an hydroxyl group in the peptidyl moiety of LP, and the absence of a double bond in the fatty acid moiety. Within subsp. *paratuberculosis* the first LP described contains a pentapeptide core, D-Phenylalanine-N-methyl-L-Valine-L-Isoleucine-L-Phenylalanine-L-Alanine, in which the N-ter D-Phenylalanine is amido-linked with a fatty acid (C18–C20) (Figure 1). This LP is termed L5P for lipopentapeptide (8), and is also known as Lipopeptide-I or Para-LP-01 (9, 10). It is an abundant molecule in *Mycobacterium avium* subsp. *paratuberculosis* (MAP) and found in the outer-most layers of the cell envelope (8, 11). Previously we deciphered the genetic organization, including the nonribosomal peptide synthase genes *mps1*, that participate in the biosynthesis of the peptidic moiety of the L5P (8). Thanks to the exact knowledge of the composition of this MAP-specific LP antigen, it was possible to produce synthetic high quality L5P in large quantity for immunological studies. Indeed, various studies by us and others described the immune response directed against this native or synthetic L5P and confirmed its potential for the detection of MAP infection (8, 10, 12–16). However, the hydrophobic nature of this LP antigen necessitates its handling and use in organic solvent, which is not suitable for industrial processes.

Despite the establishment of control programs in most developed countries, with substantial financial efforts, the prevalence rate of paratuberculosis remains at a very high level, around 50% for European herds (17, 18) and around 80% in the USA. The disease controls implemented may be different depending on the country: control, surveillance, certification, border quarantine, and on-farm biosecurity (19). For routine screening, the majority of analyses are performed by ELISA serological tests. The bovine antibody response to MAP changes over the course of disease progression. Serial bleeds collected from experimentally infected calves over a 1-year period was performed to temporally examine the humoral immune response using a MAP protein array (20). Antibody reactivity declined between day 194 and day 321 for a group of 11 proteins while reactivity to other proteins increased over the experimental timepoints. As disease enters the clinical stage, the antibody response is more consist and predominant, which enables reliable detection by ELISA (20). Currently available serological diagnostic tests are based on the use of whole cell antigens that may cross-react with closely related mycobacteria and require culture of this slow growing pathogen to produce the antigen. Specificity of commercial tests require a pre-absorption step with antigens of *M. phlei* to remove cross-reactivity. The preparation of antigen from the culture of mycobacteria is a critical element that can be a bottleneck for these tests. Conversely, pure synthetic antigens make it possible to secure and standardize the production of specific MAP antigen at low cost, thus improving existing tests. In this study we produced, by chemical synthesis, a hydrosoluble variant of L5P termed L5P-Aqueous (L5P-Aq) and evaluated these compounds for the serological diagnosis of MAP from well-defined serum banks. We also included sera from animals infected with *M. avium* subsp. *hominissuis*, a species close to MAP, and *M. bovis* because of the interest in antigens that do not cross-react with this species.

## Materials and Methods

### Chemical Synthesis of the Lipopentapeptide Antigen and Hydrosoluble Analog

The antigens were synthesized manually on a solid phase using Fmoc chemistry. The lipopentapeptide L5P was obtained using a 4-hydroxymethylbenzoyl resin (HMBA-AM resin, Novabiochem) as previously described (8).

The L5P-Aq antigen was prepared by attaching *N*-(Fmoc-13-amino-4,7,10-trioxa-tridecayl)-diglycolic acid (Novabiochem) to a Wang resin (Novabiochem) using 1-(mesitylene-2-sulfonyl)-3-nitro-1,2,4-triazole and *N*-methylimidazole (21). The capping, coupling and deprotection steps were performed as previously described (8). The product was cleaved from the resin with aqueous trifluoroacetic acid (TFA)/triisopropylsilane/H_2_O 95/2.5/2.5 v/v/v for 2 h at room temperature. After filtration of the resin, the filtrate was concentrated, and diluted with CH_2_Cl_2_/H_2_O 50/50. The organic phase was extracted twice with H_2_O. The aqueous phases were pooled and lyophilized. The crude L5P-Aq was purified by reverse-phase (RP) flash chromatography using a gradient of H_2_O+0.1%TFA/CH_3_CN+0.1%TFA from 70/30 to 50/50 and 126 mg of the peptide derivative were obtained (yield 88%). The purified compound was analyzed by RP high performance liquid chromatography (HPLC) using an Agilent 1200 pump system with a UV detector at 220 nm. A Kromasil C18 column (5 μm, 100Å, 4.6 mm × 250 mm) (Agilent, France) was used, and a gradient of acetonitrile (A)/water + 0.1%TFA (B) was applied over a period of 20 min, from 33 to 50% A (1 mL/min, retention time 10.1 min). L5P-Aq was also characterized by electrospray ionization mass spectrometry (MS) (positive mode, Q-Tof Micro Waters), quantitative amino acid analysis (AAA) (after hydrolysis with 6N HCl at 110°C for 48 h and using a Beckman 6300 analyzer), and nuclear magnetic resonance (NMR) (Bruker 400 MHz instrument). The NMR spectra and the corresponding structural formula are shown in Supplementary Figure 1.

MS: C_47_H_73_N_7_O_12_ *m/z* 928.538 [M+H]^+^ (calcd 928.540), 950.510 [M+Na]^+^ (calcd 950.522).

AAA: Ala 1 (1), Phe 1.79 (1), Ile 0.90 (1), and an extra peak typical of *N*-Methyl-Val.

^1^H NMR (MeOD): δ 0.68 (d, 3H, CH_3_γ Val, *J* = 6.56 Hz), 0.79 (d, 3H, CH_3_γ Val, *J* = 6.64 Hz), 0.81 (d, 3H, CH_3_γ Ile, *J* = 6.89 Hz), 0.85 (t, 3H, CH_3_δ Ile, *J* = 7.38 Hz), 1.01–1.09 (m, 1H, 1CH_2_γ Ile), 1.30 (d, 3H, CH_3_β Ala, *J* = 7.12 Hz), 1.45–1.51 (m, 1H, 1CH_2_γ Ile), 1.70–1.81 (m, 5H, CHβ Ile, CH_2_ D, and K), 2.08–2.14 (m, 1H, CH_2_β Val), 2.92 (dd, 1H, 1CH_2_β Phe), 3.01 (dd, 1H, 1CH_2_β Phe), 3.05 (s, 3H, NCH_3_), 3.13 (dd, 1H, 1CH_2_β Phe), 3.20 (dd, 1H, 1CH_2_β Phe), 3.23 (t, 2H, CH_2_ C or L, *J* = 6.86 Hz), 3.33 (t, 2H, CH_2_ C or L, *J* = 6.84 Hz), 3.48–3.54 (m, 4H, CH_2_ E and J), 3.56–3.64 (m, 8H, CH_2_ F, G, H, and I), 4.04 (s, 2H, CH_2_ B), 4.06–4.10 (m, 1H, CHα Ile), 4.18 (s, 2H, CH_2_ A), 4.23–4.28 (q, 1H, CHα Ala), 4.47 (d, 1H, CHα Val, *J* = 10.96 Hz), 4.61 (dt, 1H, CHα Phe), 4.68 (dt, 1H, CHα Phe), 7.16–7.19 (m, 2H, NH PEG), 7.21–7.38 (m, 10H, 2Ph), 7.97 (d, NH Ile), 8.13 (d, NH Phe). ^13^C NMR (MeOD): δ 11.34 (CH_3_δ Ile), 15.75 (CH_3_γ Ile), 18.34 (CH_3_β Ala), 19.87, 20.00 (2CH_3_γ Val), 26.10 (CH_2_γ Ile), 28.50 (CHβ Val), 30.35, 30.38 (CH_2_ D and K), 32.05 (NCH_3_), 37.69, 37.90 (CH_2_ C and L), 38.14, 38.61 (2CH_2_α Phe), 50.56 (CHα Ala), 53.35, 55.93 (2CHα Phe), 59.50 (CHα Ile), 64.94 (CHα Val), 69.22 (CH_2_ A), 69.82, 70.11 (CH_2_ E and J), 71.28, 71.31, 71.52, 71.58 (CH_2_ B, F, G, H, and I), 127.87, 129.08, 129.56, 130.27, 130.35, 130.59, 135.26, 138.28 (Ph), 171.06, 171.92, 172.20, 172.85, 173.25, 173.63, 174.43 (CO).

### Animal Sera

A bank of serum samples of bovine origin, constituted by CERVACODA, was provided by Dr. M. Govaerts. The status of the sera was characterized by several diagnostic tests: fecal culture, Pourquier ELISA, and ID-Vet ELISA. The negative sera came from farms known to be free from paratuberculosis, this status was awarded after 3 consecutive years of whole-herd negative serology. Sera were defined as being true positive (*n* = 60) when they were positive in all three tests or as true negative (*n* = 50) when they were negative in all three tests. The same serum bank was used for the discovery of new antigenic candidates in previous reports (22, 23). The sera were stored at −20°C and verified in IDEXX test (IDEXX Paratuberculosis Screening Ab, Montpellier, France) (Supplementary Tables 1, 2 and Supplementary Figure 2). In addition, a panel of sera were collected from cattle from a herd that was free of paratuberculosis but included in an official eradication campaign for bovine tuberculosis. Eleven sera were collected from cattle from which *M. bovis* had been isolated and confirmed by IS*6110* PCR and spoligotyping (24).

In the context of ovine paratuberculosis, 54 serum samples were carefully selected (25, 26). Fifteen negative sera were from unexposed sheep and 39 positive sera were from sheep experimentally infected with Telford 9.2 MAP strain (25). The positive sera, verified by commercial test (Supplementary Table 1 and Supplementary Figure 3) were classified in five groups according to the histopathological stage of the infection (27), (1) exposed but uninfected, (2) infected with low grade lesions, (3) paucibacillary 3a, (4) multibacillary 3b, and (5) macroscopical lesions 3c.

Serum samples collected from unexposed goats (*n* = 25) or goats experimentally infected either with MAP (*n* = 27) strain JII-1961 (28, 29) or with *Mycobacterium avium* subsp. *hominissuis* (MAH) field isolate (*n* = 26) (30, 31) were provided by Dr. Heike Koehler. Infection with MAP or MAH was confirmed by cultural isolation of the respective strains from tissues after necropsy and/or by histopathology (lesions) and immunohistochemistry (presence of mycobacteria) of tissue sections. Positive sera were from animals with severe lesions that tested positive in commercial diagnostic ELISA IDEXX (IDEXX Paratuberculosis Screening Ab, Montpellier, France).

### ELISA Procedure

ELISA using IDEXX commercial test (IDEXX Paratuberculosis Screening Ab, Montpellier, France) were performed according to manufacturer's instructions. ELISA experiments using synthetic antigens were performed as described previously (8). Briefly, L5P antigens were diluted at 25 μg/mL in ethanol. Water-soluble L5P-Aq variant was diluted at 80 μg/mL in PBS (10 mM PBS, pH 7.4). Maxisorp microtiter plates (Nunc, Roskilde, Denmark) were coated with 50 μL of antigen preparation. Microtiter plates coated with L5P were air-dried for 18 h at 37°C to allow the solvent to evaporate. Microtiter plates coated with L5P-Aq were incubated for 18 h at 4°C. Following each incubation step, the plates were washed three times with PBS supplemented with 0.1% (v/v) Tween 20 (PBST) (Sigma). After washing, the wells were blocked with 50 μL of Blocking Buffer (PBSTG: PBS-−0.05% Tween 20, 0.5% Gelatin) (Gibco) at room temperature for 2 h. Fifty microliters of serum (diluted at 1:100 in PBSTG) were then added to each well and incubated for 1 h 30 min at 37°C. Microtiter plates were washed five times with PBST and 50 μL of solution of Recombinant Protein G Peroxidase Conjugated (Thermo Scientific) diluted at 0.5 μg/mL in PBST, were added to each well and incubated for 1 h at room temperature. After washing five times with PBST the reaction was visualized using 3,3′,5,5′-tetramethylbenzidine (Sigma) and 0.5% v/v H_2_O_2_. The reaction was stopped with 1N sulfuric acid after 2 min of incubation in the dark and ODs were read at 450 nm on a Multiskan RC reader (Labsystems, Helsinki, Finland).

### Data Analysis

The ELISA results of positive sera compared to negative sera control, for each antigen were subjected to receiver–operator characteristic (ROC) curve analysis. This method estimates the sensitivity and specificity of the ELISA, calculate likelihood ratios and provides an overall measure of test accuracy as area under the ROC curve (AUC) to evaluate the performance of the tests (32, 33). The optimal cut-off value is provided by ROC analysis as being associated with the maximal likelihood ratio.

Statistical differences between groups of goats unexposed or goats experimentally infected either with MAP or MAH or between groups of cattle naturally infected by *M. bovis* or Map was evaluated by Wilcoxon's matched pairs test. Differences were considered significant at *p* < 0.05. All statistical analyses were performed using statistical software (GRAPHPAD PRISM version 6.07 for Windows, GraphPad software, San Diego, CA, http://www.graphpad.com).

## Results

### Chemical Synthesis of Pure L5P and Hydrosoluble Analog L5P-Aq

The L5P is very hydrophobic and does behave very differently as compared to conventional protein antigens which are hydrophilic. It is soluble in DMSO, CHCl_3_, CH_2_Cl_2_, MeOH, and EtOH, but insoluble in water or aqueous buffers. Glass containers have to be used, and contact with polypropylene surfaces must be minimized. Material handling like dilution and transfer steps also needs to be minimized. These properties of L5P would cause difficulties if a diagnostic test was based on the L5P antigen. To circumvent these difficulties, we have designed a hydrosoluble derivative of L5P, L5P-Aq. The N-ter non-immunogenic lipidic moiety of L5P was suppressed and the C-ter methyl ester was replaced by polyethylene glycol chain ending with a carboxyl group (Figure 1A).

The resulting L5P-Aq was obtained by solid-phase peptide synthesis using 9-fluorenylmethoxycarbonyl chemistry. After cleavage from the resin, by contrast with its hydrophobic counterpart, the crude product was actually extracted in aqueous phase. Purification by reverse-phase chromatography then afforded 126 mg of L5P-Aq with a 88 % overall yield. To ensure both its purity and its identity, the L5P-Aq was analyzed by RP-HPLC (Figure 1B, >99.5%), MS (Figure 1C), quantitative AAA, and NMR (Supplementary Figures 1A,B).

### Antigenic Evaluation of L5P and L5P-Aq on a Bank of Bovine Sera

**Figure 2:**
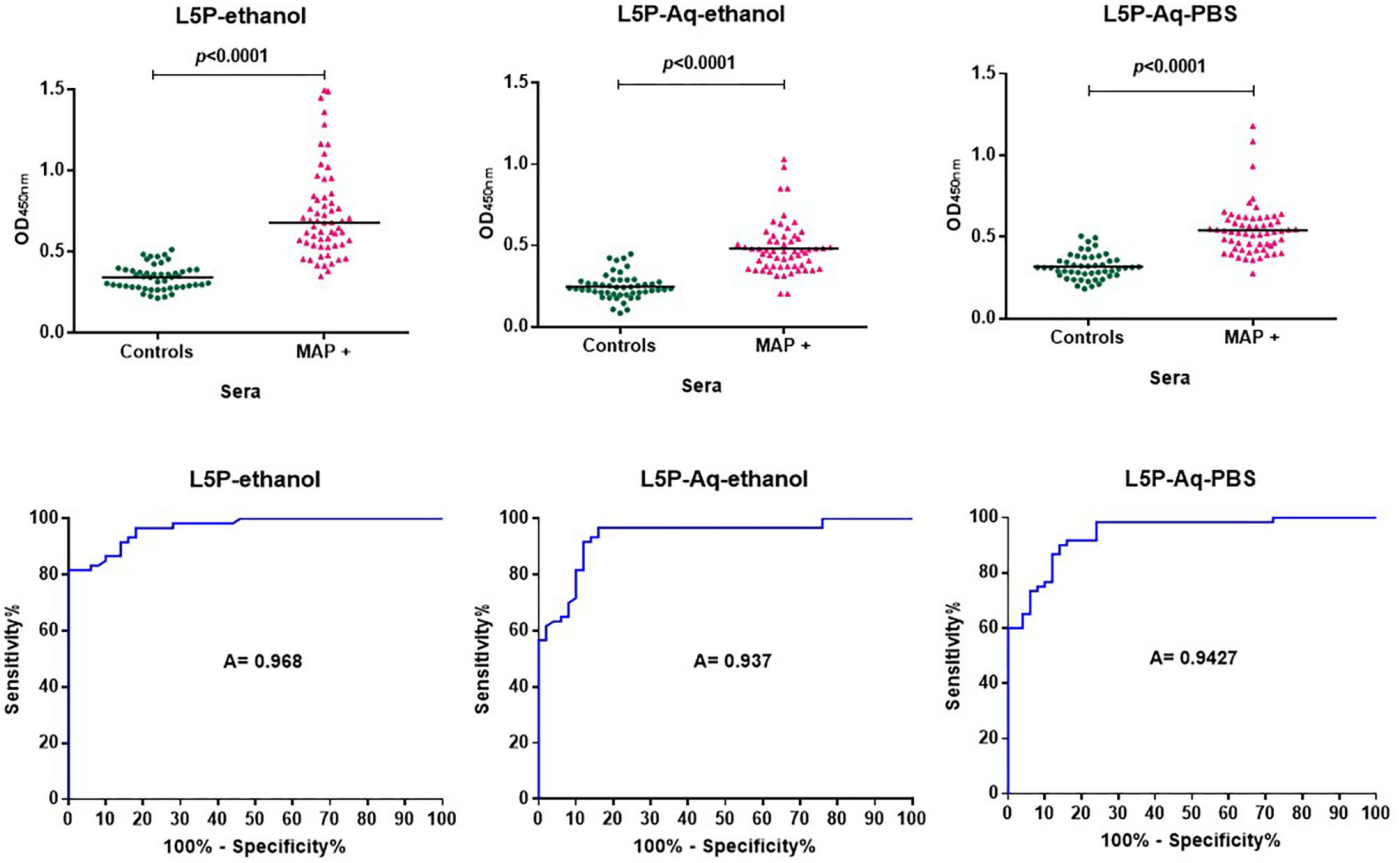
ROC analysis of antibody response of bovine sera against L5P and it hydrosoluble variant L5P-Aq. Analysis performed on a bank of sera including 60 MAP positive (MAP +) and 50 control animals, using a L5P coated in ethanol, L5P-Aq in ethanol or L5P-Aq in PBS. All results are expressed as individual OD and were compared by ROC analysis. Serum samples were tested in triplicate. The horizontal bars indicate median. A, Area under the receiver operating characteristic curve. Significantly different when *p* < 0.05.

In previous studies, the antibody response against L5P was investigated with a limited panel of sera and it has not been subjected to comprehensive, large scale evaluation. Synthetic L5P and the hydrosoluble derivative L5P-Aq were therefore both assessed using a bank of well-defined sera including 60 sera from MAP-naturally infected cattle and 50 sera from healthy cattle (details in the method section). In receiver operating characteristic analysis (ROC analysis) of L5P, the area under the curve and its standard error were 0.97 (95% confidence interval, 0.9418 to 0.9942) and 0.01336, respectively (Figure 2 and Supplementary Table 1). The L5P-Aq was evaluated in ethanol and in PBS and the area under the curve and its standard error were 0.937 (95 % confidence interval, 0.8909 to 0.9831) and 0.02354, and 0.9427 (95% confidence interval, 0.9021 to 0.9832) and 0.02068, respectively (Figure 2 and Supplementary Table 1). The sensitivity of L5P and L5P-Aq in comparison to commercial test were 81.67, 61.67, and 98%, respectively. The specificity of L5P and L5P-Aq were 98% identical to the commercial test. When tested in same conditions, the acidic derivative of the L5P lipid moiety (eicosanoic acid) was not antigenic, showing that the serum response was not directed against the L5P lipid (Supplementary Figure 4) according to previous report. These results confirm that both L5P and L5P-Aq have potential to discriminate MAP infected and uninfected cattle at a population level.

### L5P Antigenic Response in the Context of Ovine Paratuberculosis

**Figure 3:**
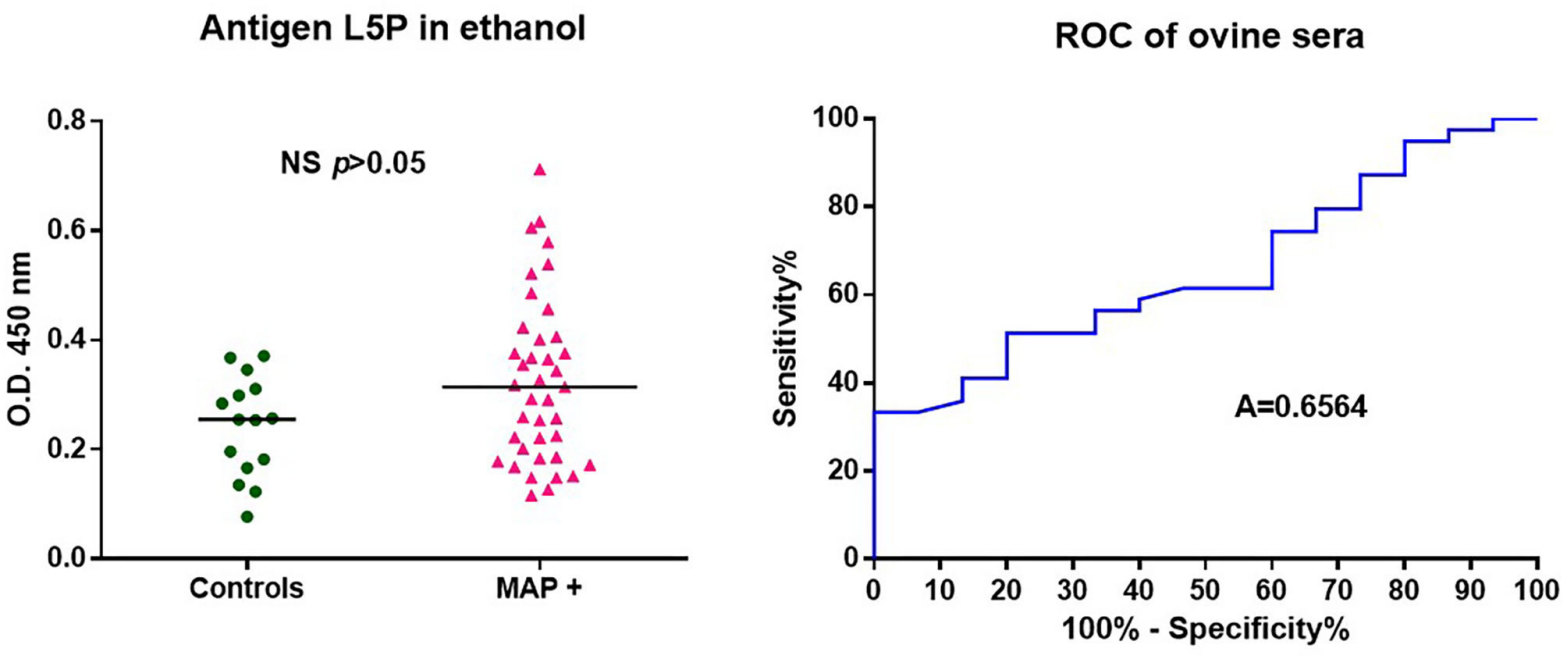
ROC analysis of antibody response of ovine sera against L5P. Analysis performed on a panel of sera from 39 MAP positive (MAP +) and 15 control animals, using L5P coated in ethanol. All results are expressed as individual OD and were compared by ROC analysis. The horizontal bars indicate median. Area under the receiver operating characteristic curve. Significantly different when *p* < 0.05. Not significantly different (NS).

Although ovine paratuberculosis is comparable to the disease in cattle, it is well-documented that MAP strains isolated from sheep have host-dependent features. To investigate the immune response in MAP infected sheep, we used the L5P described as the native antigen in strains of MAP of subtype II isolated from cattle. The results presented in Figure 3 show that synthetic L5P is not able to significantly discriminate MAP positive sheep from uninfected sheep. As discussed in detail below, this result is likely due to the minor lipopeptide differences present in the cell walls of sheep vs. cattle strains of MAP.

### Diagnostic Value of L5P in Goats Infected With MAP or MAH

**Figure 4:**
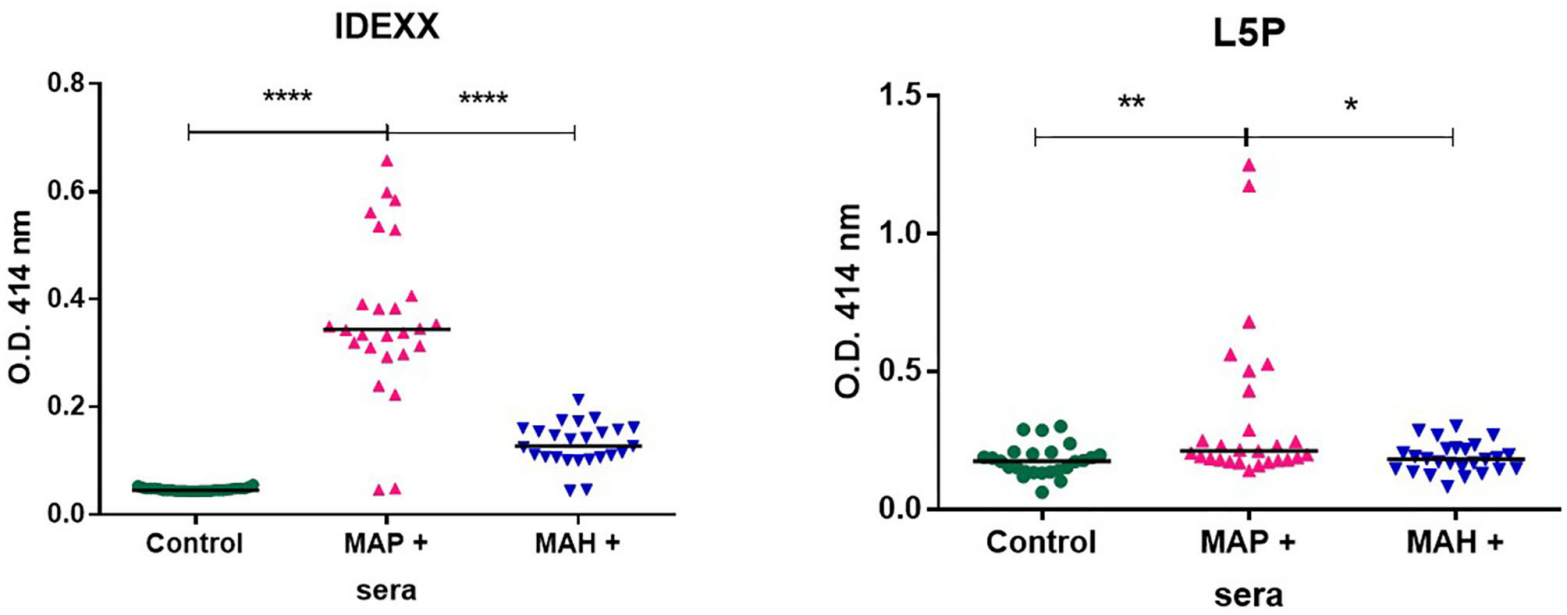
Antibody response from goats uninfected (*n* = 27) or experimentally infected with MAP (*n* = 25) or MAH (*n* = 26), as analyzed by IDEXX test or using L5P antigen. Results are shown as OD450 nm of individual samples and medians are indicated as a black line. The statistical differences between the groups were determined using the non-parametric Mann–Whitney test. Significantly different when *p* < 0.05. *p < 0.05, **p < 0.01, and ****p < 0.0001.

In their environment animals are naturally exposed to different mycobacteria. In the context of farm animals, it is recognized that this exposure can hamper diagnostic tests. To gain specificity, commercial serologic screening tests for MAP use a serum adsorption step with antigens prepared from an environmental bacterium *Mycobacterium phlei*. Knowing that L5P is specific to MAP we investigated its use without any pre-adsorption step against serum from animals experimentally infected with MAP, or MAH mycobacteria very close to MAP. As expected, the commercial IDEXX test makes it possible to distinctly detect animals infected with MAP from animals infected with MAH and from non-infected animals. Compared to the commercial test, L5P used alone without a pre-adsorption step with *Mycobacterium phlei* extracts, lacked sensitivity with MAP positive sera but didn't react with sera of animals infected with MAH (Figure 4).

### L5P as a Biomarker Able to Discriminate MAP From/Between *M. bovis* Infection

**Figure 5:**
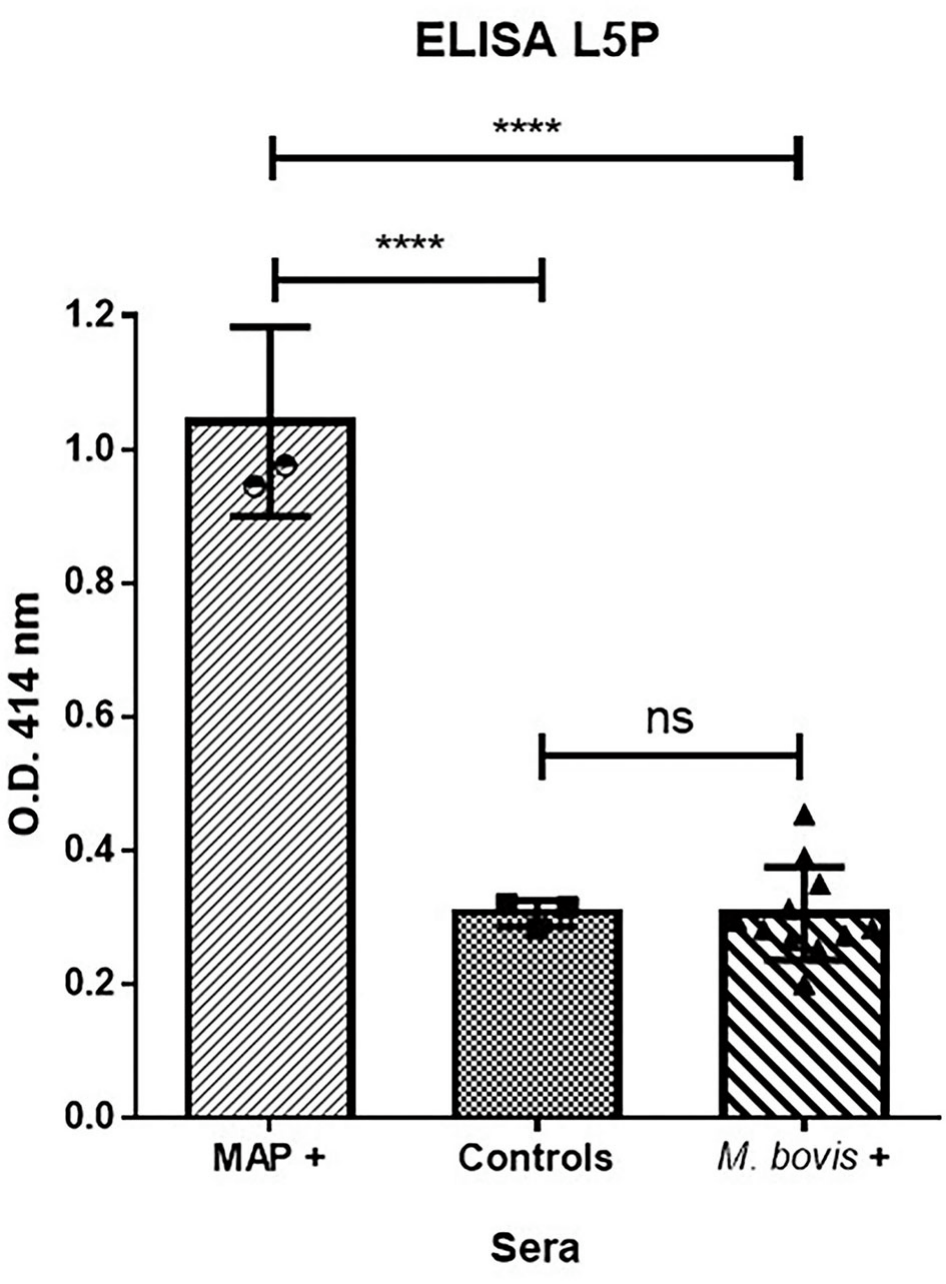
L5P used to discriminate MAP infected cattle from *M. bovis* infected cattle. ELISA were performed on plates coated with L5P in ethanol. The panel of sera tested included 3 MAP positive sera of reference, 3 negative controls, and 11 *M. bovis* positive sera. Serum samples were tested in triplicate. The statistical differences between the groups were determined using the non-parametric Mann–Whitney test. Significantly different when *p* < 0.05. ****p < 0.0001. Not significantly different (ns).

Bovine tuberculosis (bTB) is a major zoonotic disease of cattle that is endemic in much of the world. The antemortem diagnostic methods currently approved for use in cattle have limitations. The intradermal tuberculin test has suboptimal sensitivity and inconsistent performance (34, 35). Nontuberculous mycobacteria (NTM) and MAP, in particular, have been repeatedly shown to interfere with the detection of *M. bovis* (36). There have been a number of projects with aims to improve the diagnosis of bTB and JD by generating specific tools that do not compromise sensitivity or specificity due to co-infections or testing regimes. We therefore evaluated the L5P antigen against field sera from cattle naturally infected with *M. bovis*. The results in Figure 5 show that ELISA based on synthetic L5P did not react to field sera from *M. bovis* positive cattle in comparison to sera from MAP positive cattle.

## Discussion

The high lipid contents of mycobacterial cells has been recognized for a long time and much effort has been devoted to the identification of the various types of lipids present, many of which are glycolipids, unique to mycobacteria (37). *M avium* isolates are characterized by their production of highly antigenic glycopeptidolipids or GPLs, which are suitable for specific serodiagnosis (6, 38). Surprisingly, subspecies *paratuberculosis* isolates produce lipopeptides instead of GPLs; these are characterized by the absence of sugars and the absence of both hydroxylation and double bonds in the fatty acid moiety (8). Attempts to develop serological assays based on the native L5P structure are problematic due to the apolar nature of the molecule and its lack of solubility in the aqueous buffers typically used in ELISA. To overcome this issue, we engineered a hydrophilic variant of the native lipopeptide, named L5P-Aq, and we validated its antigenicity using a bank of MAP positive bovine sera. Used alone without a pre-adsorption step, the L5P ELISA test developed in this study can discriminate MAP infected goats from MAH infected goats. Interestingly, the sera from MAP infected sheep did not react with L5P, suggesting that lineage of strains of MAP specific for the sheep enrolled in this study may have another lipopeptide which has not yet been reported. This report also shows that L5P may have utility as a reagent to assist in the diagnosis of bTB.

Attempts to overcome the apolar nature of antigens in serological assays have been performed on bacterial glycolipids (39). For example, the Tween detergent has been used to optimize the antigen coating. On the other hand, the use of Tween was shown to be problematic as the detergent interacts with the lipid portion of the molecule causing its detachment from the plastic (39). From our results, 100% ethanol or methanol solutions enabled the efficient coating of antigen onto microtiter plate wells. Both our past and current studies show that the critical L5P epitopes are located on the peptide portion (8). Therefore, modifications to improve solubility were focused on (i) suppressing the hydrophobic lipid and (ii) extending the peptide with a hydrophilic chain. The resulting modification of L5P will yield numerous advantages. The hydrosolubility of the resulting L5P-Aq is an important benefit for its use in an ELISA test, especially for high throughput formats. Furthermore, safety issues associated with the use of organic solvents, including alcohols, are avoided. Finally, handling of antigenic material is much easier and reliable allowing repeatable procedures.

In most countries, the majority of routine screening for MAP (control, surveillance, certification, control at introduction) (99%) is carried out by serological ELISA tests (18, 40). The other methods such as PCR, bacteriological culture, direct examination (Ziehl-Neelsen staining) are used for clinical case confirmation. The final purpose of this project was to know if synthetic lipopeptide antigen derivatives are able to specifically discriminate sera from MAP-infected and uninfected cattle in a serological assay. Until now L5P (hydrophobic) was evaluated in just a few animals. We have thus assembled here a large panel of thoroughly characterized sera, to test the recognition of these synthetic antigens. ROC analysis of results obtained with the L5P and its hydrosoluble variant, L5P-Aq, demonstrated a cut-off value corresponding to a relative sensitivity of 82 and 62% and a specificity of 98 and 98%, respectively (Supplementary Table 1 and Supplementary Figure 2). These results show that synthetic L5P antigen, used alone, is able to discriminate MAP-infected animals and controls at a population level although the lower sensitivity compared with the commercial test remains an issue to improve. Likewise, the lack of sensitivity of L5P observed with sera from experimentally infected goats suggests that there is a “technical barrier” to be overcome to improve this parameter. Indeed to achieve the best assay performances, more research is needed to improve not only on the ELISA procedure (antigen presentation, buffer composition, coating process, secondary reagent…), but also on the target antigen(s). It is accepted that one universal antigen probably does not exist and that an efficient diagnostic test for paratuberculosis will require a cocktail of antigens. In this context, if used within an optimal combination, such synthetic individual MAP antigens have potential to assist in the improvement of the antigens used commercially. In addition, the use of synthetic antigen rather than crude protein extracts or culture-derived antigens has the advantage of avoiding the culture of slow growing pathogenic mycobacteria, such as MAP. The L5P-Aq synthesis can be easily performed by organic chemistry that offers the possibility of having large quantities of pure material. Numerous other advantages also come from the synthetic production of antigen, including the ability to standardize the batches and also to modify the antigens and/or their formulation, for example to graft fluorescent markers to monitor their handling. In the future, it would be interesting to have access to a bovine serum panel built to cover all the stages of JD infection and to be able to investigate the early stages of infection with these synthetic antigens. Preliminary data on the use of L5P for the early diagnosis of MAP infection by detection of interferon should be consolidated (15).

A very interesting issue of this study came from the “negative” result obtained with the sera from MAP infected sheep. Our current knowledge on the composition of lipids of the external layer of the wall of the subspecies *paratuberculosis* allows us to understand this result. According to genomic analysis, the S-type strains, which are more adapted in sheep, have evolved in two distinct subtype S-I and S-III (41). In a recent report we described that the SIII-type strain S397, produced a unique lipid, a tripeptide Phe-N-Methyl-Val-Ala with a N-ter lipid moiety, termed lipotripeptide (L3P) instead of the L5P detected in cattle strains (11). In addition, at the present time no L5P nor L3P has been detected in the lipids extracted from the S-I strain Telford that was used in the experimental sheep infections (data not shown). These observations may explain why L5P was not significantly recognized by the sera of infected sheep. While most MAP strains isolated from bovine are of C-type, infections with S type do occur, a situation which cannot be detected by this antigen if it is used alone. These results highlight the great need to characterize the antigens of MAP broadly across many strains, regardless of their protein or lipidic nature, and to consider the genetic diversity of this subspecies. Not only are lipids different among MAP strain types, but they can change based on the environment. For example, the L5P lipid was shown to be absent in MAP exposed to milk, but present in MAP cultured in Middlebrook media (42). Therefore, additional experiments should be conducted to determine the extent to which L5P is produced across many different environments, including feces and milk. However, from a bovine diagnostics standpoint, it is clear that MAP replicating inside cows do produce L5P as we and others have shown a bovine antibody response to L5P.

In conclusion, using an engineered synthetic antigen, we have identified a potent hydrosoluble mimic of the native L5P and established ELISA conditions for the specific diagnosis of bovine paratuberculosis. Ongoing research into the characterization of the non-protein antigens produced naturally by the different genetic lineages of MAP should identify new antigens that could contribute to achieve a diagnostic test with optimal sensitivity and specificity. In addition, it would be important to identify the L5P homolog in SI-type strains. New developments, including chemical tailoring and formulation of these synthetic antigens need to be investigated to gain sensitivity observed in this report. Other investigations should evaluate animal responses according to the disease progression and excretory status of the animal. These synthetic antigens could also be useful for the improvement of existing commercial tests especially regarding the strong demand from diagnostic laboratories for no batch-to-batch variation and elimination of the pre-absorption step.

## Data Availability Statement

The original contributions presented in the study are included in the article/Supplementary Material, further inquiries can be directed to the corresponding author/s.

## Author Contributions

SB, RW, HK, JB, and FB conceived and designed the study. All authors made substantial contributions to the analysis and writing of the manuscript.

## Conflict of Interest

The authors declare that the research was conducted in the absence of any commercial or financial relationships that could be construed as a potential conflict of interest.

## Supplementary Material

Supplementary Figure 1 ^1^H **(A)** and ^13^C **(B)** NMR spectra of L5P-Aq. The corresponding structural formula is depicted in panel **(C)**.

Supplementary Figure 2 ROC analysis of antibody response of a bank of bovine sera in the IDEXX test. ROC analysis was performed on a bank of sera including 60 MAP positive and 50 control animals, using the commercial test IDEXX. All results are expressed as individual OD and were compared by ROC analysis. Serum samples were tested in triplicate. Significantly different when *p* < 0.05. ^****^*p* < 0.0001. The horizontal bars indicate median. Not significantly different (ns).

Supplementary Figure 3 ROC analysis of antibody response of the ovine serum panel in the IDEXX test. ROC analysis performed on a bank of sera including 39 MAP positive and 15 control animals, using the commercial test IDEXX. All results are expressed as individual S/P (OD sample-OD negative control/ meanOD positive control—Mean negative control) and were compared by ROC analysis. Serum samples were tested in triplicate. Area under the receiver operating characteristic curve (A).

Supplementary Figure 4 ROC analysis of antibody response of bovine sera against lipid part of L5P. ROC analysis was performed on a bank of sera including 60 MAP positive and 50 control animals, using the lipid part of L5P coated in ethanol. All results are expressed as individual OD and were compared by ROC analysis. Serum samples were tested in triplicate. The horizontal bars indicate median. Not significantly different (ns).

Supplementary Table 1 Statistical detail of ROC analysis.

Supplementary Table 2 Detail of bank of sera.
