# Supplementary figures and images for: Engineering Synthetic Lipopeptide Antigen for Specific Detection of Mycobacterium avium subsp. paratuberculosis Infection
Source: Front Vet Sci. 2021 Apr 23;8:637841. doi: 10.3389/fvets.2021.637841 (PMC8103206; doi:10.3389/fvets.2021.637841)

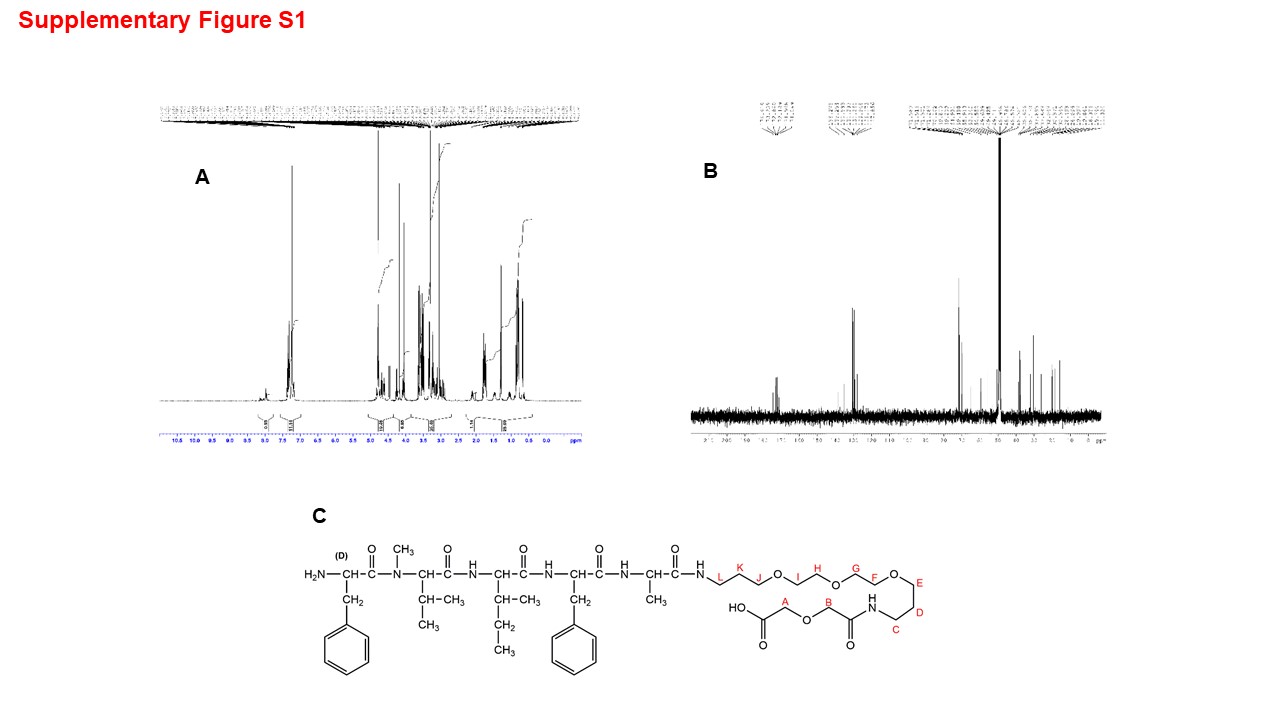

Supplement: Supplementary Figure 1 — 1H (A) and 13C (B) NMR spectra of L5P-Aq. The corresponding structural formula is depicted in panel (C). [file Image_1.JPEG]

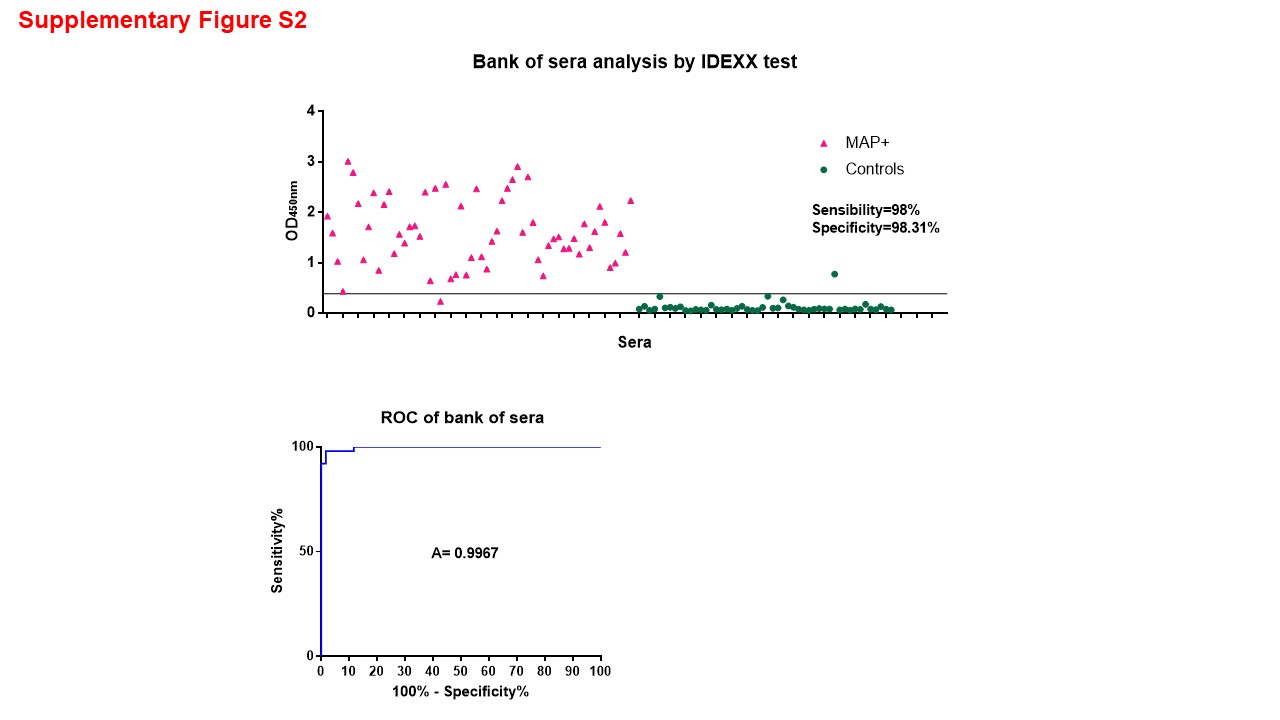

Supplement: Supplementary Figure 2 — ROC analysis of antibody response of a bank of bovine sera in the IDEXX test. ROC analysis was performed on a bank of sera including 60 MAP positive and 50 control animals, using the commercial test IDEXX. All results are expressed as individual OD and were compared by ROC analysis. Serum samples were tested in triplicate. Significantly different when p < 0.05. ****p < 0.0001. The horizontal bars indicate median. Not significantly different (ns). [file Image_2.JPEG]

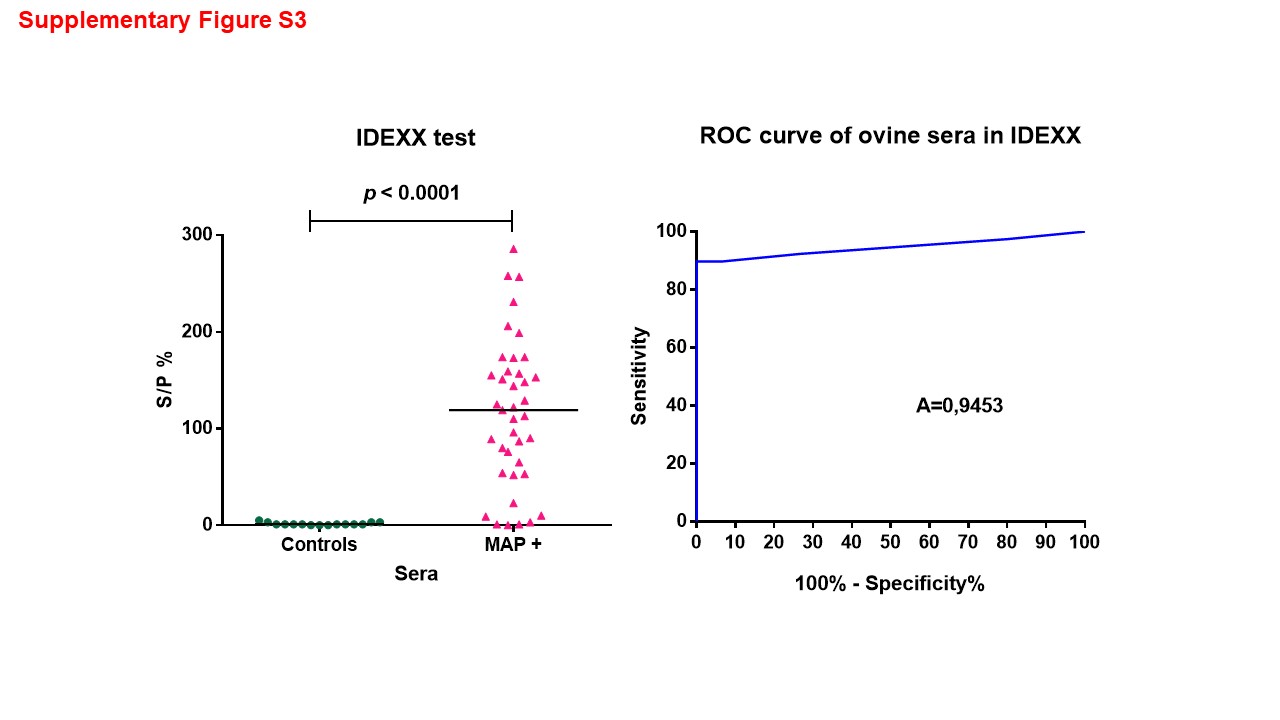

Supplement: Supplementary Figure 3 — ROC analysis of antibody response of the ovine serum panel in the IDEXX test. ROC analysis performed on a bank of sera including 39 MAP positive and 15 control animals, using the commercial test IDEXX. All results are expressed as individual S/P (OD sample-OD negative control/ meanOD positive control—Mean negative control) and were compared by ROC analysis. Serum samples were tested in triplicate. Area under the receiver operating characteristic curve (A). [file Image_3.JPEG]

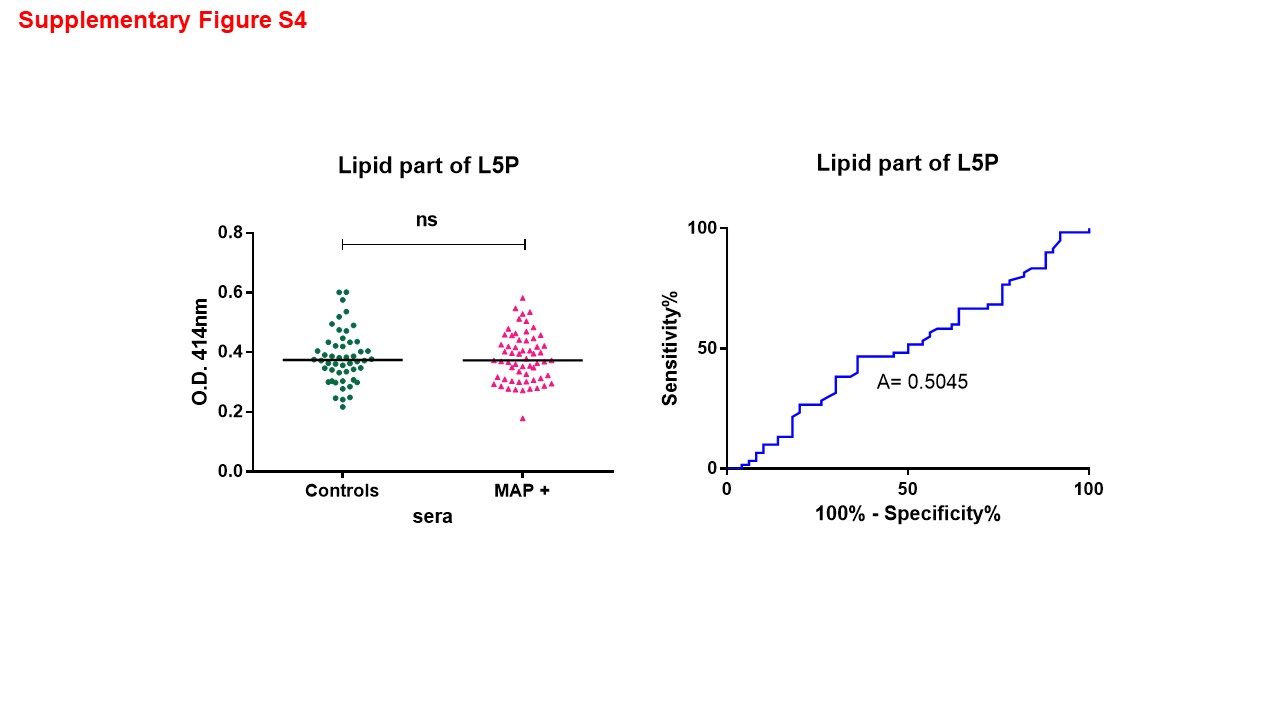

Supplement: Supplementary Figure 4 — ROC analysis of antibody response of bovine sera against lipid part of L5P. ROC analysis was performed on a bank of sera including 60 MAP positive and 50 control animals, using the lipid part of L5P coated in ethanol. All results are expressed as individual OD and were compared by ROC analysis. Serum samples were tested in triplicate. The horizontal bars indicate median. Not significantly different (ns). [file Image_4.JPEG]
